# Supplementary material for: Ig-VAE: Generative modeling of protein structure by direct 3D coordinate generation
Source: PLoS Comput Biol. 2022 Jun 27;18(6):e1010271. doi: 10.1371/journal.pcbi.1010271 (PMC9269947; doi:10.1371/journal.pcbi.1010271)
Supplement: S1 File — mov format of the interpolation movie for the trajectory analyzed in Fig 4. interpolation.mp4. mp4 format of the interpolation movie for the trajectory analyzed in Fig 4. patchdock_output_I.pdb. The raw patchdock output used for design I in Fig 5. patchdock_output_II.pdb. The raw patchdock output used for design II in Fig 5. S1 Methods. Detailed documentation of model training, architecture, and all design protocols used in the study. supplemental_methods_attachment.zip. Scripts and commands used for design. Referenced by supplemental_methods_rev.pdf. (ZIP) [file pcbi.1010271.s001.zip › S1_File/S1_Methods.pdf]

Supplemental Methods

1 Additional Model Information

The input dimensions of the model are 512×512 corresponding to four the backbone atoms (N, C_α, C, O) over 128 residues. The output dimensions of the model are 512×3 corresponding to the 3D coordinates of the same atoms. The IgVAE architecture is fully convolutional, with an 11-layer encoder, and a 15-layer decoder. All down-sampling operations in the encoder were done with a convolution operation, while all up-sampling operations in the decoder were done with transposed convolutions. At each layer LeakyReLU was used as the non-linearity with a "leak" parameter of 0.1. The size of the latent embedding was 1024.

2 Loss Function

We adopt the following notation:

$$\begin{aligned} x &: \text{Coordinate Data} \\ z \sim N(0, I) &: \text{Latent Vector} \\ q_\theta &: \text{Encoder} \\ p_\phi &: \text{Decoder} \\ x' = p_\phi(q_\theta(x)) &: \text{Reconstructed Coordinates} \\ z_x = q_\theta(x) &: \text{Latent Embedding of } x \end{aligned}$$

The loss function can be written as:

$$L_{\theta,\phi} = \frac{1}{n_{\text{data}}} \sum_{x \in \text{data}} ReconLoss_{\theta,\phi}(x) + \lambda \; KL(q_\theta(z|x)||p(z))$$

The reconstruction loss is comprised of distance matrix ($Dist$) and torsion($Tors$) components:

$$ReconLoss_{\theta,\phi}(x) = w_{dist} \; Dist(x, x') + w_{tors} \; Tors(x, x')$$

$Tors$ is computed as the L_2 distance between the unit sphere projections of the real and reconstructed backbone torsion angles. $Dist$ is comprised of three terms, each of which are L_2 losses between different components of the real and reconstructed distance matrices. All distance matrices in the loss include all backbone atoms in the order: N, C_α, C, O.

$$Dist(x, x') = w_{pad} \; Pad(x, x') + w_{local} \; Local(x, x') + w_{nonlocal} \; NonLocal(x, x')$$

Pad denotes the loss incurred by the padded regions, $Local$ denotes the loss incurred by atoms that are within 4 residues of one another, and $NonLocal$ denotes the loss incurred at all other positions. The weights of the loss function are annealed according to the following schedule:

Weight	$\text{Value}_{\text{Iterations}}$
λ	$1.0e^{-4}_{150K}$, $5.0e^{-4}_{150K}$, $1.0e^{-3}_{150K}$, $2.5e^{-3}_{150K}$, $7.5e^{-3}_{150K}$, $1.0e^{-2}_{150K}$, $1.5e^{-2}_{1.02\text{M}}$
w_{dist}	$0.1_{1.92\text{M}}$
w_{tors}	20.0_{870K}, $1.0_{1.05M}$
w_{pad}	$1.0e^{-3}_{915K}$, $1.0e^{-4}_{350K}$, 0.0_{655K}
w_{local}	2.0_{100K}, 2.5_{100K}, 3.0_{100K}, 3.5_{100K}, $4.0_{1.52M}$
w_{nonlocal}	$1.0_{1.92M}$

3 Data Preparation

All of the structures in the dataset were idealized and relaxed before use. The relax step was performed with constraints to starting coordinates. The idealization and relax commands are included as run_ideal.sh and run_relax.sh respectively. Training set structures that were smaller than 128 residues were "structurally padded" by appending residues to both ends of the molecule to maintain centering. Padding was applied using RosettaRemodel, with an example blueprint and flags included as pad.bp and pad.sh respectively.

4 Structural Cropping

During generation the model is allowed to generate a full array of cartesian coordinates for 512 atoms (128 residues) spanning the maximum output size of our model. However, in most cases the generated structure will be smaller than 128 residues with unused generated atoms at the terminal regions disconnected and randomly placed in space. These disconnected atoms can be easily identified by eye and removed. However, to automate the process, we perform a nearest-neighbor structural alignment using Cα distance matrices, which allows for quick identification of the beta-strand regions and removal of the excess atoms.

5 Refinement and Design

5.1 Centroid Refinement

In the specified experiments, centroid refinement was performed using Rosetta FastRelax in cartesian mode with constraints to the starting coordinates. FastRelax was run via the RosettaScripts scripting interface. The script is included as cent_refine.xml, flags are included as run_cent_refine.sh, and the scoring weights are included as cen_std_cart.wts.

5.2 Sequence Design

Sequence design for individual domains was performed using Rosetta FastDesign[1] via the RosettaScripts[2] interface using the ref2015[3] score function. The design scripts are included as design.xml and run_design.sh. Generated models were passed thorough a constrained centroid refinement step (see above) before design.

6 Computational Binder Design

6.1 Backbone Selection

To design the SARS-CoV2-RBD binders, we first generated 5000 backbones by random sampling, and then performed constrained centroid refinement. Structures with a post-refinement energy greater than -0.870 per residue were removed from the set, leaving 4311 structures remaining. All of these were run though PatchDock[4] against the CoV2-RBD with both the binder and target handled as poly-valine backbones. This was done to select for structures with high shape complementarity in a sequence-agnostic way. PatchDock was run with the package-default settings, and was chosen specifically for this step because it is known to place heavy weight on molecular shape complementarity. The top 20 outputs from each PatchDock run were collected (86220 structures), and ranked based on the proportion of loop residues contacting the ACE2 epitope. The ACE2 epitope was determined based on contacting residues in PDB:6VW1, using a C_β distance cutoff of 5 Å. 2 backbones from among the top 10 were manually selected for sequence design. PatchDock was accessed on June 28, 2020. All PatchDock outputs are available upon request, and PatchDock outputs for designs I and II in Figure 5A are provided in the Supplemental Materials.

6.2 Interface Sequence Design

Sequence design of the bound complex was performed in two steps. In the first step, design was done with a softened repulsive penalty via the ref2015_soft score weights, and backbones were constrained to their starting coordinates. In the second step, we performed 10 rounds of design, with each round consisting of soft-repulsive design, normal design, and minimization. Between each round, the new designs were accepted or rejected based on ddG and solvent accessible surface area (SASA) improvements. No coordinate constraints were applied in the second step. The scripts for the first design step are included as interf_design1.xml and run_interf_design1.sh. The scripts for the second design step are included as interf_design2.xml and run_interf_design2.sh.

6.3 Docking

Validation of the designed interfaces was performed using RosettaDock[5, 6] with 100000 decoys per run. All of the docking runs were global, and were performed with the documentation-recommended settings (https://tinyurl.com/yy3mgufp). The exact run flags are included as run_dock.sh.

References

[1] Gaurav Bhardwaj, Vikram Khipple Mulligan, Christopher D. Bahl, Jason M. Gilmore, Peta J. Harvey, Olivier Cheneval, Garry W. Buchko, Surya V. S. R. K. Pulavarti, Quentin Kaas, Alexander Eletsky, Po-Ssu Huang, William A. Johnsen, Per Jr Greisen, Gabriel J. Rocklin, Yifan Song, Thomas W. Linsky, Andrew Watkins, Stephen A. Rettie, Xianzhong Xu, Lauren P. Carter, Richard Bonneau, James M. Olson, Evangelos Coutsias, Colin E. Correnti, Thomas Szyperski, David J. Craik, and David Baker. Accurate de novo design of hyperstable constrained peptides. *Nature*, 538(7625):329–335, October 2016.

[2] Sarel J. Fleishman, Andrew Leaver-Fay, Jacob E. Corn, Eva-Maria Strauch, Sagar D. Khare, Nobuyasu Koga, Justin Ashworth, Paul Murphy, Florian Richter, Gordon Lemmon, Jens Meiler, and David Baker. RosettaScripts: A Scripting Language Interface to the Rosetta Macromolecular Modeling Suite. *PLoS ONE*, 6(6):e20161, June 2011.

[3] Rebecca F. Alford, Andrew Leaver-Fay, Jeliazko R. Jeliazkov, Matthew J. O'Meara, Frank P. DiMaio, Hahnbeom Park, Maxim V. Shapovalov, P. Douglas Renfrew, Vikram K. Mulligan, Kalli Kappel, Jason W. Labonte, Michael S. Pacella, Richard Bonneau, Philip Bradley, Roland L. Dunbrack, Rhiju Das, David Baker, Brian Kuhlman, Tanja Kortemme, and Jeffrey J. Gray. The Rosetta All-Atom Energy Function for Macromolecular Modeling and Design. *Journal of Chemical Theory and Computation*, 13(6):3031–3048, June 2017.

[4] D. Schneidman-Duhovny, Y. Inbar, R. Nussinov, and H. J. Wolfson. PatchDock and SymmDock: servers for rigid and symmetric docking. *Nucleic Acids Research*, 33(Web Server):W363–W367, July 2005.

[5] Sidhartha Chaudhury, Monica Berrondo, Brian D. Weitzner, Pravin Muthu, Hannah Bergman, and Jeffrey J. Gray. Benchmarking and Analysis of Protein Docking Performance in Rosetta v3.2. *PLoS ONE*, 6(8):e22477, August 2011.

[6] Jeffrey J. Gray, Stewart Moughon, Chu Wang, Ora Schueler-Furman, Brian Kuhlman, Carol A. Rohl, and David Baker. Protein–Protein Docking with Simultaneous Optimization of Rigid-body Displacement and Side-chain Conformations. *Journal of Molecular Biology*, 331(1):281–299, August 2003.
