# Supplementary figures and images for: Ig-VAE: Generative modeling of protein structure by direct 3D coordinate generation
Source: PLoS Comput Biol. 2022 Jun 27;18(6):e1010271. doi: 10.1371/journal.pcbi.1010271 (PMC9269947; doi:10.1371/journal.pcbi.1010271)

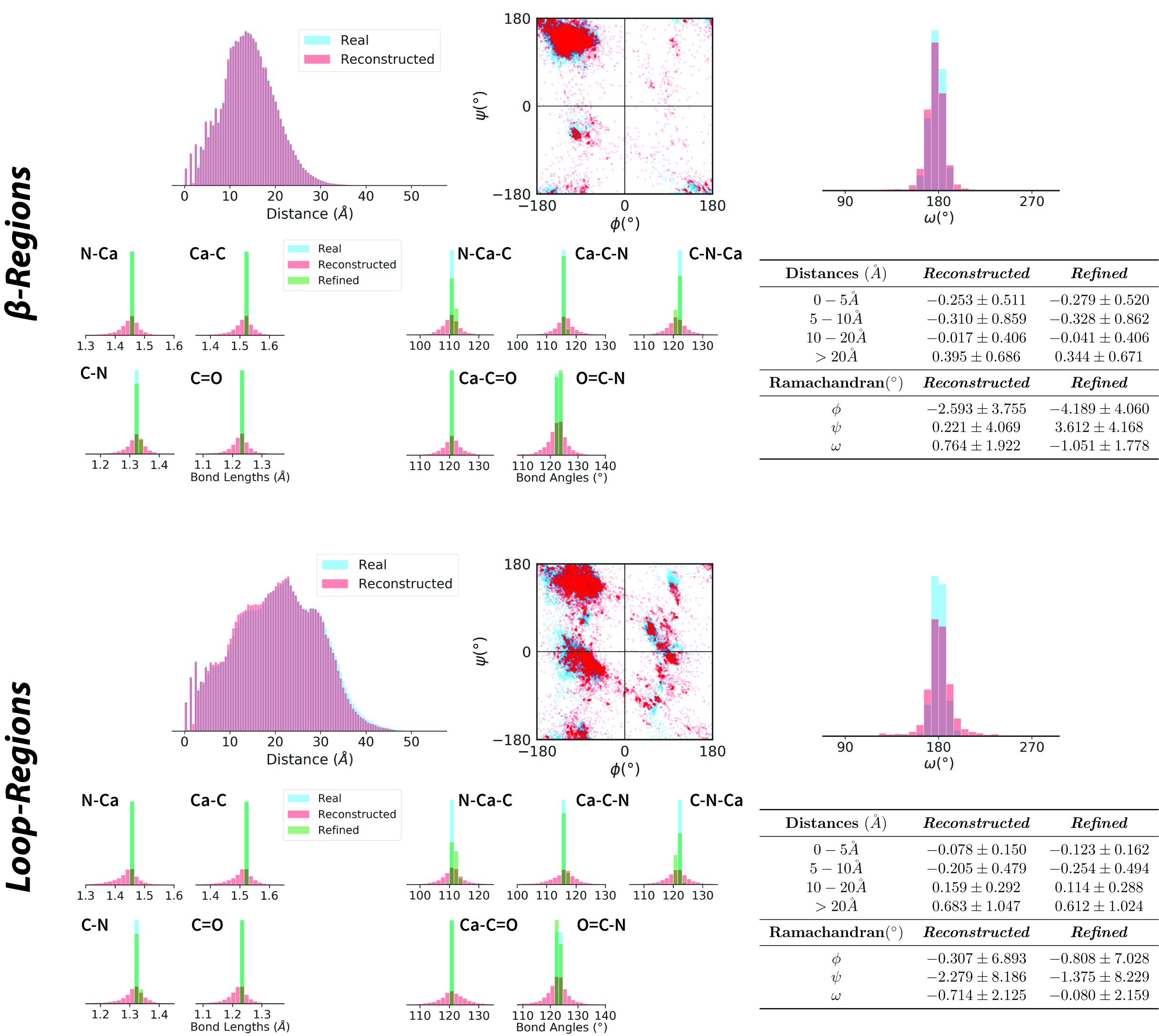

Supplement: S1 Fig — Reconstruction data for 500 randomly chosen, non-redundant structures in the training set, plotted separately for the beta and loop regions. The top row in each section shows, overlays of the pairwise distance and Ramachandran distributions of the real and reconstructed data. The bottom rows show overlays of the bond length and bond angle distributions of the real, reconstructed and refined data. Real data are shown in blue, reconstructed in pink, and refined in green. In the bottom right of each section we show a table of the reconstruction errors in pairwise distance and Ramachandran angle before and after refinement. The distance errors are reported as per-pairwise-distance error averaged over all structures in the dataset, and analogously for the angle errors. (TIF) [file pcbi.1010271.s002.tif]

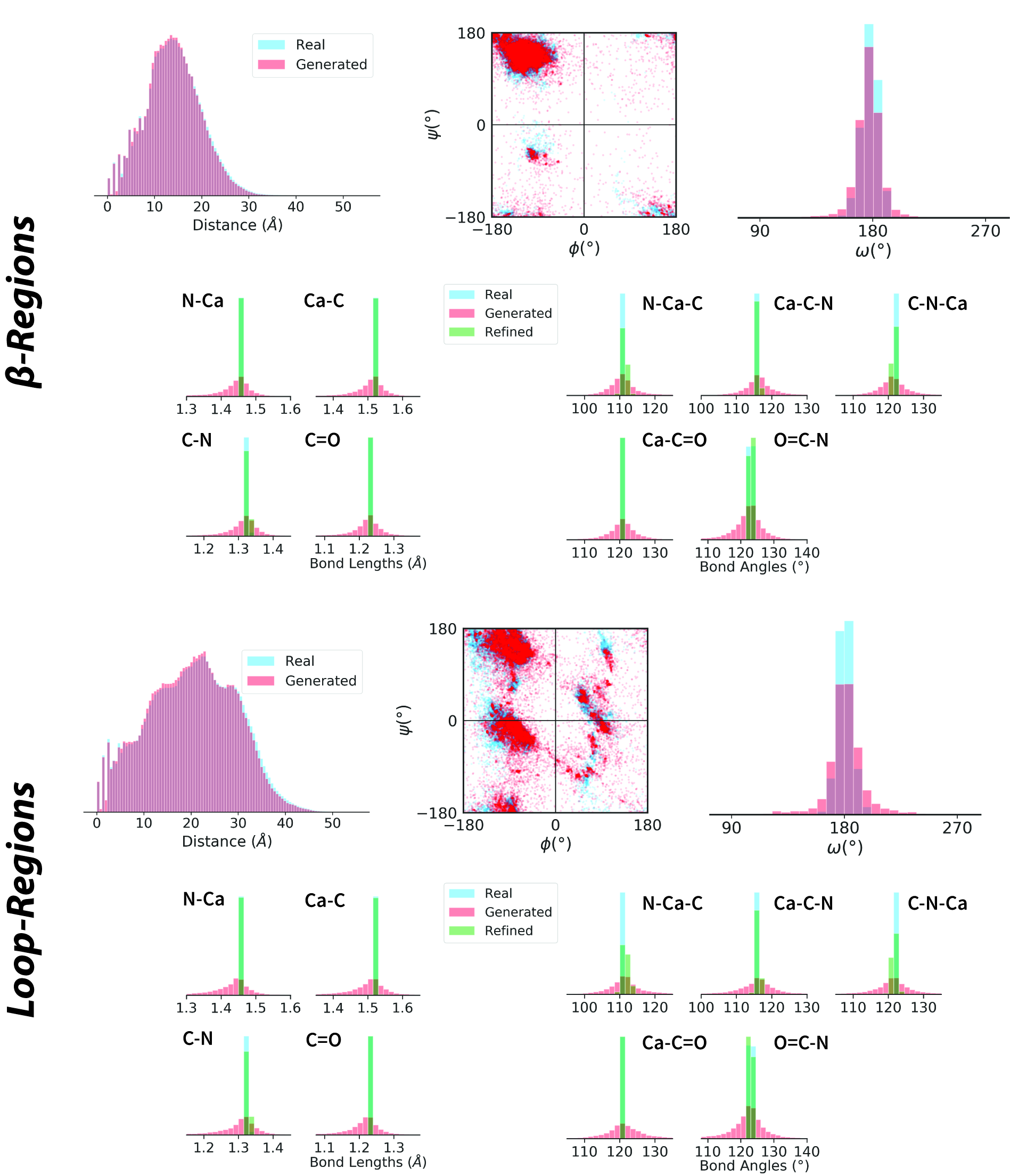

Supplement: S2 Fig — Data for 500 of randomly selected non-redundant training samples and generated structures, plotted separately for the beta and loop regions. The top row in each section shows, overlays of the pairwise distance and Ramachandran distributions of the real and reconstructed data. The bottom rows show overlays of the bond length and bond angle distributions of the real, reconstructed and refined data. Real data are shown in blue, reconstructed in pink, and refined in green. (TIF) [file pcbi.1010271.s003.tif]

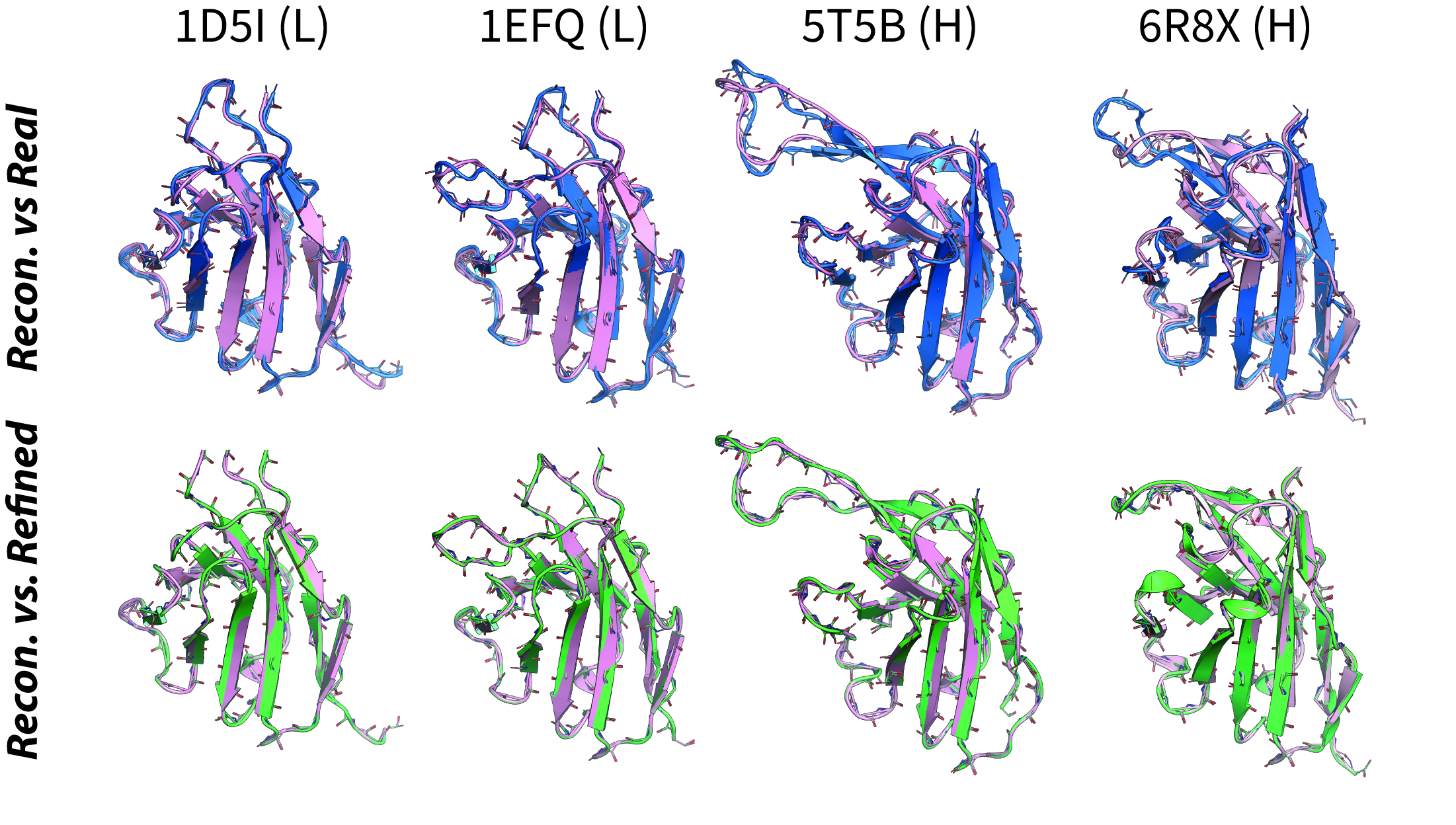

Supplement: S3 Fig — A higher resolution rendering of the reconstruction examples shown in Fig 2C for two heavy-chain and two light-chain examples. Real structures are shown in blue, reconstructed structures are shown in pink, and refined structures are shown in green. (TIF) [file pcbi.1010271.s004.tif]

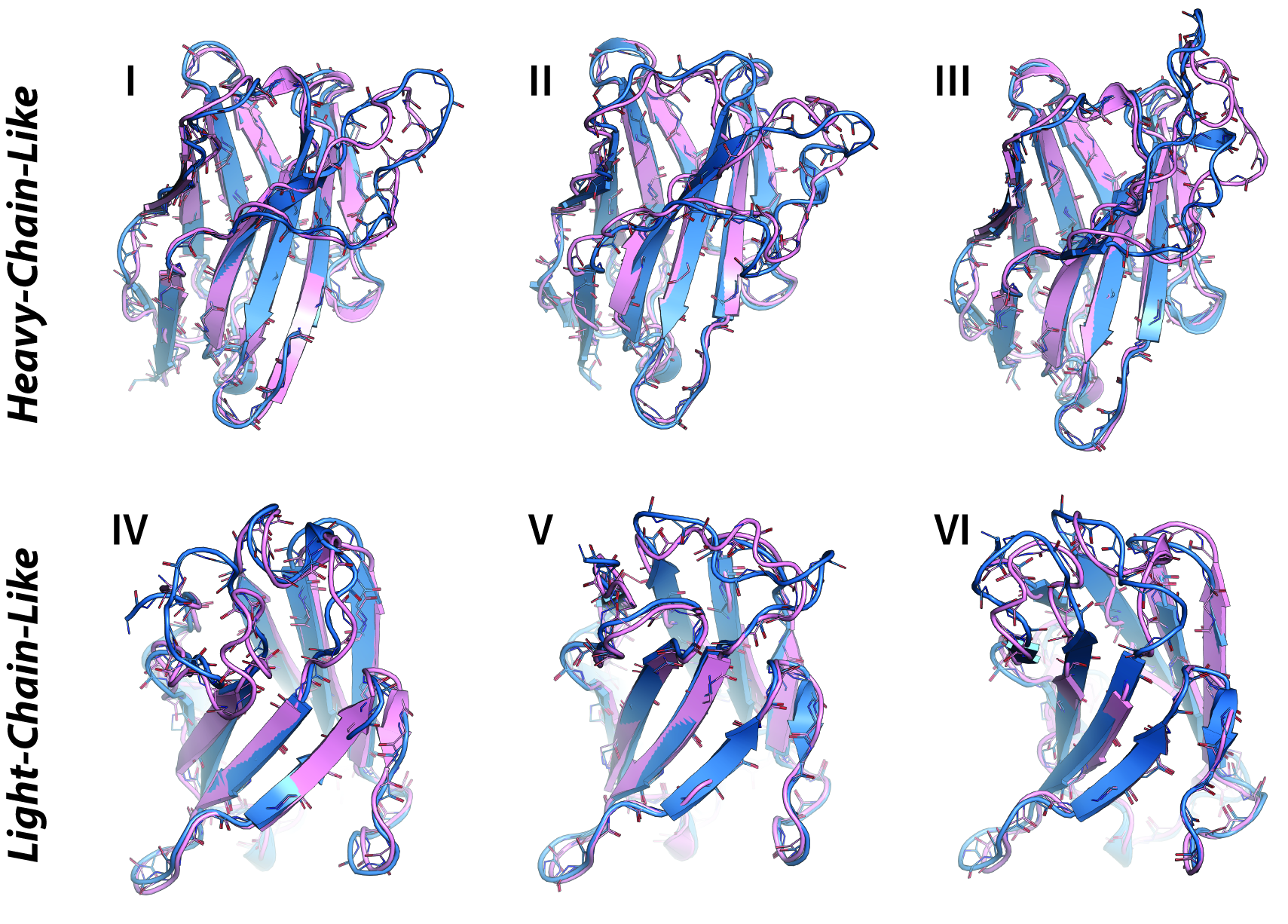

Supplement: S4 Fig — A higher resolution rendering of the generated examples shown in Fig 3D (Center), selected based on the analysis in Fig 3D (Left), for two heavy-chain and two light-chain examples. Real structures are shown in blue, reconstructed structures are shown in pink, and refined structures are shown in green. Each alignment shows an overlay of the generated structures (pink) and their nearest neighbors (blue) in the training set. The roman numerals correspond to the same structures as in Fig 3D. (TIF) [file pcbi.1010271.s005.tif]
